# Role of Diet Quality in the Association Between Excess Weight and Psychosocial Problems in a Large Sample of Children in Spain

**DOI:** 10.1001/jamanetworkopen.2022.9574

**Published:** 2022-04-29

**Authors:** José Francisco López-Gil, Iván Cavero-Redondo, Estela Jiménez-López, Bruno Bizzozero-Peroni, Alicia Saz-Lara, Arthur Eumann Mesas

**Affiliations:** 1 Health and Social Research Center, Universidad de Castilla–La Mancha, Cuenca, Spain; 2 Department of Psychiatry, Hospital Virgen de La Luz, CIBERSAM, Cuenca, Spain; 3 Instituto Superior de Educación Física, Universidad de la República, Rivera, Uruguay; 4 Postgraduate Program in Public Health, Universidade Estadual de Londrina, Londrina, Paraná, Brazil

## Abstract

**Importance:**

The role of healthy eating in the association between excess weight and psychosocial health among children is unknown.

**Objective:**

To investigate whether a diet based on healthy eating habits moderates the association between excess weight and psychosocial problems in a large sample of children in Spain.

**Design, Setting, and Participants:**

This population-based cross-sectional study used data on a representative sample of 3772 children collected in 2017 through the Spanish National Health Survey. Statistical analysis was conducted from September 21 to October 27, 2021.

**Main Outcomes and Measures:**

Body mass index was calculated as weight in kilograms divided by height in meters squared; body mass index *z* scores were computed following the sex and age criteria of the International Obesity Task Force and were used to determine excess weight. Diet quality was assessed using the Spanish Healthy Eating Index (S-HEI; a higher score denotes greater adherence to the guidelines of the Spanish Society of Community Nutrition and, therefore, a higher quality of diet). Psychosocial problems were assessed with the Strengths and Difficulties Questionnaire completed by parents or guardians. All analyses were adjusted for major confounders.

**Results:**

Among the 3772 participants (1908 boys [50.6%]; mean [SD] age, 9.5 [3.1] years), the prevalence of excess weight (ie, overweight or obesity) was 38.4% (n = 1448). Diet quality moderated the association between excess weight and psychosocial problems (β = –0.06; 95% CI, –0.09 to –0.02). Moderation analysis revealed 2 different regions of significance according to the S-HEI score. First, the association between excess weight and psychosocial problems was greater for children with an S-HEI score lower than 67.5. Second, the association of excess weight with psychosocial problems was lower for children with an S-HEI score higher than 84.9. In addition, a neutral area was found, indicating that the association between excess weight and psychosocial problems neither increased nor decreased in those with an S-HEI score between 67.5 and 84.9.

**Conclusions and Relevance:**

The results of this study suggest that the association between excess weight and psychosocial problems is moderated by diet quality among children in Spain. These findings are clinically relevant because psychosocial problems are a major concern among children with excess weight. Because obesity is a chronic disease, it requires ongoing counseling and treatment throughout life.

## Key Points

**Question:**

What is the role of healthy eating habits in the association between excess weight and psychosocial problems in a large sample of children in Spain?

**Findings:**

This cross-sectional study of 3772 children in Spain found that diet quality plays a moderating role in the association between excess weight and psychosocial problems.

**Meaning:**

This study suggests that for a large sample of children in Spain, the association between excess weight and psychosocial problems is moderated by diet quality.

## Teaser

This cross-sectional study investigates whether a diet based on healthy eating habits moderates the association between excess weight and psychosocial problems in a large sample of children in Spain.

## Introduction

Psychosocial health is broadly defined as including psychological and social psychological outcomes that are interrelated with socioeconomic factors. However, there is no consensus on this definition in the field.^1^ Most psychosocial problems, such as emotional or behavioral problems, begin in childhood,^2^ have been shown to be relatively stable over time,^3^ and may be the cause of years lived with a disability.^4^ Therefore, the prevention of psychosocial problems during childhood and adolescence through early detection and early intervention is crucial^2^ to improving long-term outcomes.^5^

Furthermore, it is well established that childhood obesity is one of the most serious public health concerns of the 21st century because of its adverse and chronic outcomes, including those associated with psychosocial health conditions.^6^ In this regard, children and adolescents with obesity are considered to be at increased risk for developing comorbid conditions associated with psychosocial health, such as depression, anxiety, low self-esteem, lower quality of life, and behavioral disorders.^7^

In addition to childhood obesity, there are multiple factors associated with the development and persistence of psychosocial health problems.^8^ Thus, it has been suggested that the greater the number of risk factors to which children and adolescents are exposed, the greater the potential effect is on their psychosocial health.^2^ In this regard, it has been shown that children who engage in unhealthy behaviors, such as being physically inactive and/or consuming low-quality food, are more likely to develop obesity and, therefore, to increase their risk for developing metabolic diseases and psychosocial problems.^9^ Concerning the association between healthy eating and psychosocial health, a systematic review conducted by Khalid et al^10^ included 20 studies on the association between diet and depression among children and adolescents and showed that an unhealthy diet, characterized by the consumption of low-quality foods, was associated with depression or psychosocial health disorders. Similarly, a systematic review by O’Neil et al^11^ that included 12 epidemiologic studies found significant evidence for a cross-sectional association between unhealthy dietary patterns and lower psychosocial health among children and adolescents. Taken together, the findings of these systematic reviews suggest that diet plays a role in the association between excess weight and psychosocial health. However, the specific circumstances under which diet quality may moderate the association between excess weight and psychosocial health are unclear. These reviews point to the need for studies based on representative samples that support greater external validity. Furthermore, they report that potential confounding variables (eg, socioeconomic status) were often not included or controlled for.

The cause of obesity is complex^12^ and involves the interaction of social,^13^ biological,^13^ genetic,^14^ and environmental factors,^13,15^ among others.^13^ Regarding environmental factors, diet quality has been shown to play a moderating role in the association between excess weight and lifestyle outcomes (eg, physical activity)^16^ or health outcomes (eg, inflammatory biomarkers).^17^ However, to our knowledge, the moderating role of healthy eating in the association between excess weight and psychosocial health among children and adolescents is unknown. Therefore, it could be hypothesized that a higher-quality diet could help to counteract the association of excess weight with psychosocial health. Thus, this study examined whether healthy eating moderates the association between excess weight and psychosocial health in a large national sample of children in Spain.

## Methods

### Population Sample and Study Design

A nationwide cross-sectional study was conducted using data from the 2017 Spanish National Health Survey.^18^ This survey was conducted by the Ministry of Health, Consumer Affairs and Social Welfare and the National Statistics Institute.^19^ The sampling framework involved noninstitutionalized individuals in Spain. A 3-stage sampling design was applied. The census section was the first stage, households were the second-stage units, and individuals were the third-stage units. Within each household, an adult (aged ≥15 years) was selected to complete the adult questionnaire, and if there were children (aged ≤14 years) in the household, 1 child was probabilistically selected from among the members to complete the minor’s questionnaire. Participants were informed of the survey method by means of a letter from the Ministry of Health, Consumer Affairs and Social Welfare that described the aims of the survey, the anonymous and voluntary nature of participation, and the visit of a qualified and authorized interviewer. This study was conducted using deidentified public domain databases,^18^ following European regulations and principles on ethics and data protection. After the children’s parents or guardians provided written informed consent, interviews with them were conducted at the household level of the general population by qualified interviewers from the Spanish National Institute of Statistics. Therefore, it was not necessary to submit the project for review by an ethics committee. This study followed the Strengthening the Reporting of Observational Studies in Epidemiology (STROBE) reporting guideline.

For this study, the sample was restricted to individuals aged 14 years or younger (those who answered the minor’s questionnaire). The original sample consisted of 6101 participants. As psychosocial problems were evaluated, only children and adolescents aged 4 to 14 years were included, and 1502 participants (24.6%) younger than 4 years were excluded. In addition, 827 participants (13.6%) were excluded owing to missing data on diet quality, weight, height, or any covariate considered in the study. Thus, the final sample included 3772 children (61.8%). The differences between excluded and included participants are shown in eTable 1 in the Supplement.

### Procedures

#### Excess Weight (Independent Variable)

Weight and height were reported by parents or guardians. These values were used to determine the body mass index (BMI), which was transformed into a BMI *z* score following the sex and age criteria of the International Obesity Task Force.^20^ According to the BMI *z* score, participants were categorized as having either “no excess weight” (thinness and normal weight) or “excess weight” (overweight and obesity).

#### Psychosocial Problems (Dependent Variable)

The Strengths and Difficulties Questionnaire (SDQ)^21^ parent version form is a widely applied measurement tool to assess different behavioral, emotional, and social problems associated with psychosocial health among young people. In this study, the Spanish version of the SDQ,^22^ which has been validated in previous studies,^23,24^ was used. The SDQ contains 25 statements distributed across 5 different subscales: (1) emotional problems, (2) conduct problems, (3) hyperactivity, (4) peer problems, and (5) prosocial behavior. A Likert scale with 3 possible options (0 = not true, 1 = somewhat true, and 2 = certainly true) is used, and the combined score for each subscale ranges from 0 to 10 points. The first 4 subscales (emotional problems, conduct problems, hyperactivity, and peer problems) were used to establish a total psychosocial problems score, with higher scores indicating poorer psychosocial health. The fifth subscale (prosocial behavior) assesses resources rather than problems and is conceptually different from the assessment of psychological difficulties. Thus, its score was not included in the total psychosocial problems score.^21^

#### Diet Quality (Potential Moderator)

Diet quality was assessed using the Spanish Healthy Eating Index (S-HEI),^25^ which is an adapted version of the original Healthy Eating Index.^26^ The S-HEI includes 10 food groups (vegetables, cereals, legumes, fruit, meat, dairy, sweets, cold meals, soft drinks, and dietary variety). Parents or guardians were asked about the frequency of consumption of each of these food groups. The following 5 response options are available: never or hardly ever; 1 time per week; once or twice per week; more than 3 times per week, but not daily; and daily. These response options are defined according to the frequency of food intake as indicated within the guidelines of the Spanish Society of Community Nutrition.^27^ The combined score for each food group ranges from 0 to 10 points. The total S-HEI score is the sum of the scores obtained for each of the food groups. A higher S-HEI score denotes greater adherence to the guidelines of the Spanish Society of Community Nutrition and, therefore, a higher quality of diet.

#### Covariates

The following variables were considered in adjusting the analyses because of their potential confounding effects on the association between obesity and psychosocial problems.^7,28^ Age, sex, region, and nativity status (born in Spain or not born in Spain) were stated by parents or guardians. Socioeconomic status was categorized according to the occupation of the reference adult in the household unit. Physical activity was evaluated with a short questionnaire adapted from the International Physical Activity Questionnaire^29^; in our study, it was limited to a single question about the performance of physical activity during the child’s leisure time. The question had 4 possible response options: (1) no exercise (free time occupied mainly by sedentary activities, such as reading, watching television, going to the cinema); (2) occasional physical activity or sport; (3) physical activity several times a month; and (4) sports or physical training several times a week.^18^ Recreational screen time was reported by the parents or guardians independently for weekdays and weekend days in answer to the following question: “How much time does your child typically spend on a weekday in front of a screen, including a computer, tablet, television, video, video game, or cell phone screen?” The possible response options were (1) no time or almost no time, (2) less than 1 hour, and (3) 1 hour or more. Sleep duration was assessed with the following question: “Can you tell me approximately how many hours your child usually sleeps daily? (Including nap times).”

### Statistical Analysis

Statistical analysis was conducted from September 21 to October 27, 2021. Descriptive data are shown as absolute and relative numbers and percentages for categorical variables and as the mean (SD) values for continuous variables. Linear regression analyses were performed to assess the association of the S-HEI score with excess weight and psychosocial problems. Moderation analyses were conducted using PROCESS macro 4.0 in IBM SPSS Statistics for Windows, version 25.0 (IBM Corp). The PROCESS macro applies ordinary least-squares analysis to estimate moderation models (model 1 in PROCESS) using psychosocial problems as the dependent variable, excess weight as the independent variable, and S-HEI score as the moderator variable, with a bootstrapping-resampling procedure (10 000 samples).^30^ For this purpose, a pick-a-point approach was performed as a method for probing moderation, including arbitrary values (ie, mean and ±1 SD). These values were categorized into low (−1 SD), medium (mean), and high (+1 SD). In addition, to eliminate the need to choose arbitrary values of the moderator when probing an interaction, the Johnson-Neyman procedure was performed to test interactions and to find regions of statistical significance and different point estimates of the moderators and confidence intervals.^31^ All analyses were adjusted for sex, age, region, nativity status, socioeconomic status, physical activity, recreational screen time, and sleep duration. A 2-tailed *P* value of less than .05 was used to establish statistical significance.

## Results

The Table shows the characteristics of the study participants.^20^ The prevalence of participants with excess weight (ie, overweight or obesity) among the 3772 participants (1908 boys [50.6%]; mean [SD] age, 9.5 [3.1] years) was 38.4% (n = 1448). The mean (SD) S-HEI score (diet quality indicator) was slightly higher among participants without excess weight (70.0 [8.8]) than among participants with excess weight (69.5 [9.3]), although the difference was not statistically significant (*P* = .08). In addition, the mean (SD) SDQ score (psychosocial problems indicator) was significantly higher among participants with excess weight (7.8 [5.2]) than it was among those without excess weight (7.2 [5.1]) (*P* = .001).

**Table.** Descriptive Data on 3772 Children in Spain

Variable	Children, No. (%)		*P* value
	No excess weight (n = 2324 [61.6%])	Excess weight (n = 1448 [38.4%])	
Age, mean (SD), y	9.6 (3.2)	9.3 (3.0)	.001
Sex			
Boys	1159 (49.9)	749 (51.7)	.27
Girls	1165 (50.1)	699 (48.3)	
Nativity status			
Born in Spain	2225 (95.7)	1366 (94.3)	.05
Not born in Spain	99 (4.3)	82 (5.7)	
Socioeconomic status			
1 (highest)	352 (15.1)	155 (10.7)	<.001
2	212 (9.1)	105 (7.3)	
3	502 (21.6)	254 (17.5)	
4	351 (15.1)	178 (12.3)	
5	675 (29.0)	524 (36.2)	
6 (lowest)	232 (10.0)	232 (16.0)	
Anthropometric data, mean (SD)			
Weight, kg	33.9 (12.3)	43.3 (16.5)	<.001
Height, cm	140.3 (19.8)	137.9 (21.9)	.001
BMI, *z* score^a^	−0.24 (1.00)	1.88 (0.73)	<.001
Psychosocial problems			
SDQ score, mean (SD)	7.2 (5.1)	7.8 (5.2)	.001
Eating healthy			
S-HEI score, mean (SD)	70.0 (8.8)	69.5 (9.3)	.08
Physical activity			
No exercise	307 (13.2)	214 (14.8)	.02
Occasional physical activity or sport	476 (20.5)	348 (24.0)	
Physical activity several times a month	729 (31.4)	422 (29.1)	
Sports or physical training several times a week	812 (34.9)	464 (32.0)	
Recreational screen time, mean (SD), min	118.4 (69.4)	121.3 (72.4)	.24
Sleep duration, mean (SD), h	558.2 (61.5)	553.1 (62.8)	.01

Abbreviations: BMI, body mass index; SDQ, Strengths and Difficulties Questionnaire; S-HEI, Spanish Healthy Eating Index.

^a^ According to the International Obesity Task Force criteria.^20^

**Figure 1.**
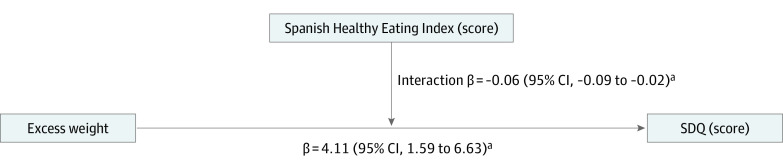
Moderation Analyses of the Role of the Spanish Healthy Eating Index in the Association Between Excess Weight and Psychosocial Problems β Coefficients are presented as standardized regression coefficients and their 95% CIs, adjusted by sex, age, region, nativity status, socioeconomic status, physical activity, recreational screen time, and sleep duration. SDQ indicates Strengths and Difficulties Questionnaire. ^a^*P* < .05.

Figure 1 depicts the moderation analysis after ordinary least-squares regression. This finding indicated a positive association between excess weight and psychosocial problems among the children (β = 4.11; 95% CI, 1.59-6.63). Moreover, this positive association of excess weight with psychosocial problems was moderated by the S-HEI score (β = –0.06; 95% CI, –0.09 to –0.02). The moderating role of the S-HEI score in the association between BMI status (eg, thinness, normal weight, overweight, or obesity) and psychosocial problems (ie, SDQ score) is shown in the eFigure in the Supplement.

**Figure 2.**
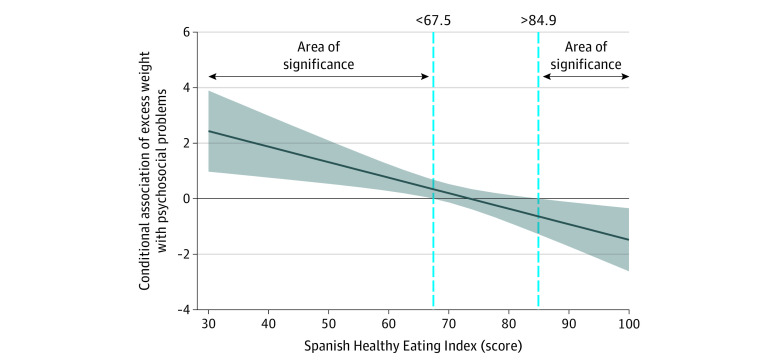
Moderating Role of Spanish Healthy Eating Index Score in the Association Between Excess Weight and Psychosocial Problems Using the Johnson-Neyman Procedure Data are shown as regression point estimates (unstandardized β coefficients) and their 95% CIs, adjusted by sex, age, region, nativity status, socioeconomic status, physical activity, recreational screen time, and sleep duration.

The moderating role of the S-HEI score in the association between weight status and psychosocial problems (SDQ score) is shown in Figure 2. The Johnson-Neyman procedure indicated different point estimates of the S-HEI score as a moderator, using the slope and the different significant regions. First, the region exposed to an S-HEI score lower than 67.5 indicates that the association between excess weight and psychosocial problems is greater for children who scored in this region. Second, the score region from 67.5 to 84.9 shows that the association between excess weight and psychosocial problems is neither increased nor decreased for those with an S-HEI score between the lower and upper thresholds. Third, the region above a score of 84.9 indicates that the association of excess weight with psychosocial problems was lower for children who scored above this cutoff point on the S-HEI. The detailed values obtained by the Johnson-Neyman technique are shown in eTable 2 in the Supplement.

## Discussion

This study explored the moderating role of diet quality in the association between excess weight and psychosocial problems in a large sample of children in Spain. Initially, the association between excess weight and a greater number of psychosocial problems was confirmed in this nationally representative sample of children and adolescents from Spain. Furthermore, the main finding of that study was that the association between excess weight and psychosocial problems was moderated by the quality of diet as assessed with the S-HEI.

The finding that excess weight was associated with the development of psychosocial problems among young people is consistent with the findings reported in a systematic review and a recent longitudinal study.^28^ Although, to our knowledge, the moderating role of diet quality in this association has not been previously examined, there is evidence of an association between excess weight and the early introduction and maintenance of inadequate diet quality as characterized by excessive consumption of energy-dense, nutrient-poor foods and insufficient consumption of nutrient-rich foods. The early introduction and persistence of these dietary behaviors may lead to the nutrient and energy excesses that are associated with excess weight^32^ and to the nutrient deficiencies that are associated with psychosocial health problems.^11^

Fundamentally, obesity is a disease of energy imbalance because a long-term positive energy balance, in which energy intake exceeds expenditure, leads to an excess of energy stored as triglycerides in adipose tissue. However, this overly simplistic characterization belies the complexity of obesity.^33^ In fact, the validity of the energy balance paradigm for obesity has recently been questioned.^12^ Obesity is characterized by an array of immunopathologies and neuropathologies (eg, leptin resistance) that perpetuate this condition.^33^ Similarly, there is also substantial evidence that epigenetic mechanisms (eg, DNA methylation and histone modification) may play a role in the development of obesity.^14^ In addition, environmental factors, such as city living, commuting, school location, physical inactivity (not practicing regular physical exercise), sedentary behavior (having large amounts of time in a lower-energy expenditure posture [eg, sitting] regardless of regular exercise), pollutants, and diet, among others, play a significant role in environment-dependent body weight dysregulation.^15^ In association with diet, excessive dietary energy intake has been associated with increased consumption of unhealthy foods, such as ultra-processed foods, which have excessive amounts of free sugars, saturated fats, calories, and sodium.^32^ In this sense, a recent systematic review by Liberali et al^34^ concluded that children and adolescents who eat an unhealthy diet (composed of obesogenic foods) are more likely to develop obesity. Moreover, eating habits may not only have an effect on physical appearance but may also be associated with lower psychosocial health.^10,11,35^ In a systematic review by O’Neil et al,^11^ most of the included studies consistently demonstrated significant associations between unhealthy dietary patterns and poorer psychosocial health when dietary patterns were examined as the exposure. Furthermore, the systematic review by Khalid et al^10^ also reported significant cross-sectional associations between unhealthy diets and depression, with effect sizes ranging from small to moderate. In similar findings but from a different perspective, a recent systematic review showed that better overall diet quality is associated with more positive psychosocial health among preadolescent children.^35^ Taken together, this evidence supports our findings that diet quality plays a key role in the association between excess weight and psychosocial problems among children.

Some possible biological pathways can be suggested to explain why obesity is associated with psychosocial health among young people under certain unhealthy dietary patterns of consumption. First, vitamin deficiencies are more likely to occur in individuals with excess weight than they are in individuals with normal weight, mainly because of insufficient consumption of healthy vitamin-rich foods (eg, fruits, vegetables, dairy products).^36^ Consequently, according to current evidence, a lack of the vitamins typically available in these healthy foods, such as B vitamins (ie, B_6_, B_9,_ and B_12_) and vitamin D, could lead to poorer cognitive performance, lower mood status, and an increased likelihood of depression.^37,38,39,40^ However, the specific pathway linking the intake of these healthy fatty acids to psychosocial health still requires further research. For example, although preliminary experimental models have shown that ω-3 fatty acid supplementation is associated with an increased concentration of the brain-derived neurotrophic factor and increased cognitive performance,^41^ these associations have not been confirmed among patients with diabetes and major depression.^42^ Finally, reduced consumption of fiber-rich foods (eg, fruits and vegetables) by children and adolescents, in addition to increasing the risk for obesity by reducing satiety and increasing snacking and total energy intake,^43^ may be associated with an increased likelihood of psychosocial problems, such as depression.^44^ This finding could be explained by the association between gut microbiota and psychosocial health because the prebiotic effect of fiber may promote improvements in the composition of the gut microbiome and reduce oxidative stress.^45^

### Limitations and Strengths

This study has some limitations and strengths. First, owing to the cross-sectional design of this study, we cannot establish whether the observed associations imply cause and effect. Moreover, reverse causation may occur if young people with psychosocial problems have excess weight, and vice versa. Second, excess weight was determined by height and weight measurements reported by parents or guardians, which could have introduced measurement error. In addition, self-reported questionnaires were used; therefore, the risk of reporting bias cannot be ruled out. In this regard, adults with obesity tend to underestimate the amount of carbohydrate-rich foods consumed by their children^46^; thus, parents or guardians with obesity may have overestimated their children’s S-HEI score. However, both the S-HEI^26^ and SDQ^21^ are validated instruments widely used in the scientific literature. Additionally, we analyzed the proposed association in a large and representative sample of children in Spain; therefore, the evidence provided is robust and has high external validity.

## Conclusions

The present findings are based on a national sample of children and indicate that excess weight is associated with a higher frequency of psychosocial problems, although this association is observed only among children with a lower-quality diet. These findings are clinically relevant because psychosocial problems are a major concern for young people with excess weight. Furthermore, these findings may have public health implications, indicating that improving diet quality could be a strategy by which to prevent psychosocial problems among children and adolescents with excess weight. However, these conclusions require confirmation in prospective studies specifically designed to assess the medium- and long-term outcomes of changes in diet quality among children at risk of developing psychosocial problems throughout their adolescence and young adulthood. Moreover, because obesity is a chronic disease, it requires ongoing counseling and treatment throughout life, even if young people have a high-quality diet and fail to lose weight.

## Notes

Supplement. **eTable 1.** Characteristics and Differences Between Study Participants Who Were Included or Not in the Final Analysis **eTable 2.** Conditional Effect of Excess Weight at Values of the Spanish Healthy Eating Index Score, Applying the Johnson-Neyman Technique **eFigure.** A Visual Representation of the Moderation of the Spanish Healthy Eating Index in the Association Between Body Mass Index Status and Psychosocial Problems
