# Supplementary material for: Role of Diet Quality in the Association Between Excess Weight and Psychosocial Problems in a Large Sample of Children in Spain
Source: JAMA Netw Open. 2022 Apr 29;5(4):e229574. doi: 10.1001/jamanetworkopen.2022.9574 (PMC9055454; doi:10.1001/jamanetworkopen.2022.9574)
Supplement: Supplement. — eTable 1. Characteristics and Differences Between Study Participants Who Were Included or Not in the Final Analysis eTable 2. Conditional Effect of Excess Weight at Values of the Spanish Healthy Eating Index Score, Applying the Johnson-Neyman Technique eFigure. A Visual Representation of the Moderation of the Spanish Healthy Eating Index in the Association Between Body Mass Index Status and Psychosocial Problems [file jamanetwopen-e229574-s001.pdf]

Supplementary Online Content

López-Gil JF, Cavero-Redondo I, Jiménez-López E, Bizzozero-Peroni B, Saz-Lara A, Mesas AE. Role of diet quality in the association between excess weight and psychosocial problems in a large sample of children in Spain. *JAMA Netw Open.* 2022;5(4):e229574. doi:10.1001/jamanetworkopen.2022.9574

eTable 1. Characteristics and Differences Between Study Participants Who Were Included or Not in the Final Analysis
eTable 2. Conditional Effect of Excess Weight at Values of the Spanish Healthy Eating Index Score, Applying the Johnson-Neyman Technique
eFigure. A Visual Representation of the Moderation of the Spanish Healthy Eating Index in the Association Between Body Mass Index Status and Psychosocial Problems

This supplementary material has been provided by the authors to give readers additional information about their work.

© 2022 López-Gil JF et al. *JAMA Network Open.*

eTable 1. Characteristics and Differences Between Study Participants Who Were Included or Not in the Final Analysis

Variables	Total sample		Excluded sample		Analyzed sample		*p*
	N	No. (%)	n	No. (%)	n	No. (%)	
Age, mean (SD), years	6106	7.6 (4.3)	2334	4.6 (4.2)	3772	9.4 (3.1)	<0.001
Sex							
Males	6106	3174 (52.0)	2334	1266 (54.2)	3772	1908 (50.6)	0.005
Females	6106	2932 (48.0)	2334	1068 (45.8)	3772	1864 (49.4)	
Nativity status							
Native-born	6106	5646 (92.5)	2334	2196 (94.1)	3772	1812 (95.0)	0.06
Foreign-born	6106	460 (7.5)	2334	138 (5.9)	3772	96 (5.0)	
SES					3772		
Status 1 (the highest)	5665	764 (13.5)	1893	257 (13.6)	3772	253 (13.3)	0.20
Status 2	5665	498 (8.8)	1893	181 (9.6)	3772	162 (8.5)	
Status 3	5665	1115 (19.7)	1893	359 (19.0)	3772	394 (20.6)	
Status 4	5665	763 (13.5)	1893	234 (12.4)	3772	265 (13.9)	
Status 5	5665	1801 (31.8)	1893	602 (31.8)	3772	592 (31.0)	
Status 6 (the lowest)	5665	724 (12.8)	1893	260 (13.7)	3772	242 (12.7)	
Anthropometric data							
Weight, mean (SD), kg	5768	31.2 (16.7)	1996	19.2 (13.2)	3772	37.3 (15.3)	<0.001
Height. mean (SD), cm	5511	127.4 (30.4)	1739	101.5 (31.2)	3772	139.0 (21.0)	<0.001
BMI, mean (SD), z-score[a]	4833	0.55 (1.70)	1061	0.47 (2.55)	3772	0.60 (1.40)	<0.001
Psychosocial problems							
SDQ, mean (SD), score	4604	7.4 (5.1)	832	7.3 (5.1)	3772	7.4 (5.1)	0.61
Eating healthy							
S-HEI, mean (SD), score	5816	70.4 (9.5)	2044	71.5 (10.1)	3772	69.5 (9.1)	<0.001
Physical activity							
No exercise	6068	1600 (26.4)	2296	324 (14.1)	3772	1276 (33.8)	<0.001
Occasional physical activity or sport	5832	4755 (81.5)	2060	1780 (86.4)	3772	2975 (78.9)	<0.001
Physical activity several times a month	5830	3514 (60.3)	2058	1496 (71.7)	3772	2018 (53.5)	<0.001
Sports or physical training several times a week	5827	3305 (56.7)	2055	1448 (70.5)	3772	2921 (77.4)	<0.001
Sedentary behavior							
Recreational screen time, mean (SD), min	5827	109.0 (72.3)	2055	89.7 (71.4)	3772	119.5 (70.6)	<0.001
Sleep							
Sleep duration, mean (SD), min	6106	4522 (74.1)	2334	629.8 (117.7)	3772	556.2 (62.1)	<0.001

Abbreviations: SD, Standard deviation; SES, Socioeconomic status; BMI, Body mass index; SDQ, Strengths and Difficulties Questionnaire; S-HEI, Spanish Healthy Eating Index. [20, a]According to the International Obesity Task Force criteria.

© 2022 López-Gil JF et al. *JAMA Network Open.*

eTable 2. Conditional Effect of Excess Weight at Values of the Spanish Healthy Eating Index Score, Applying the Johnson-Neyman Technique[a]

S-HEI mean (score)	Effect	SE	*t*	*p*	LLCI	ULCI
29.0	2.49	0.76	3.267	0.001	1.00	3.98
32.7	2.28	0.70	3.278	0.001	0.92	3.64
36.5	2.07	0.63	3.290	0.001	0.84	3.31
40.2	1.86	0.56	3.302	0.001	0.76	2.97
43.9	1.65	0.50	3.311	0.001	0.67	2.63
47.7	1.44	0.44	3.315	0.001	0.59	2.30
51.4	1.24	0.37	3.307	0.001	0.50	1.97
55.2	1.03	0.31	3.267	0.001	0.41	1.64
58.9	0.82	0.26	3.155	0.002	0.31	1.33
62.6	0.61	0.21	2.873	0.004	0.19	1.03
66.4	0.40	0.18	2.237	0.03	0.05	0.75
**67.5**	**0.34**	**0.17**	**1.961**	**0.05**	**0.00**	**0.68**
70.1	0.19	0.17	1.136	0.26	-0.14	0.52
73.8	-0.02	0.18	-0.095	0.92	-0.38	0.34
77.6	-0.23	0.22	-1.022	0.31	-0.66	0.21
81.3	-0.44	0.27	-1.607	0.11	-0.97	0.10
**84.9**	**-0.64**	**0.33**	**-1.961**	**0.05**	**-1.28**	**0.00**
85.1	-0.64	0.33	-1.969	0.05	-1.29	0.00
88.8	-0.85	0.39	-2.203	0.03	-1.61	-0.09
92.5	-1.06	0.45	-2.361	0.02	-1.94	-0.18
96.3	-1.27	0.51	-2.474	0.01	-2.28	-0.26
100.0	-1.48	0.58	-2.557	0.01	-2.61	-0.35

Abbreviations: S-HEI, Spanish Healthy Eating Index; SE, standard error; LLCI, lower limit confidence interval; ULCI, upper limit confidence interval.

[a]Bold indicates the statistically significant cut-off point of the moderator variable. Adjusted by sex, age, region, nativity status, socioeconomic status, physical activity, recreational screen time and sleep duration.

© 2022 López-Gil JF et al. *JAMA Network Open.*

eFigure. A Visual Representation of the Moderation of the Spanish Healthy Eating Index in the Association Between Body Mass Index Status and Psychosocial Problems. Adjusted by sex, age, region, nativity status, socioeconomic status, physical activity, recreational screen time and sleep duration.

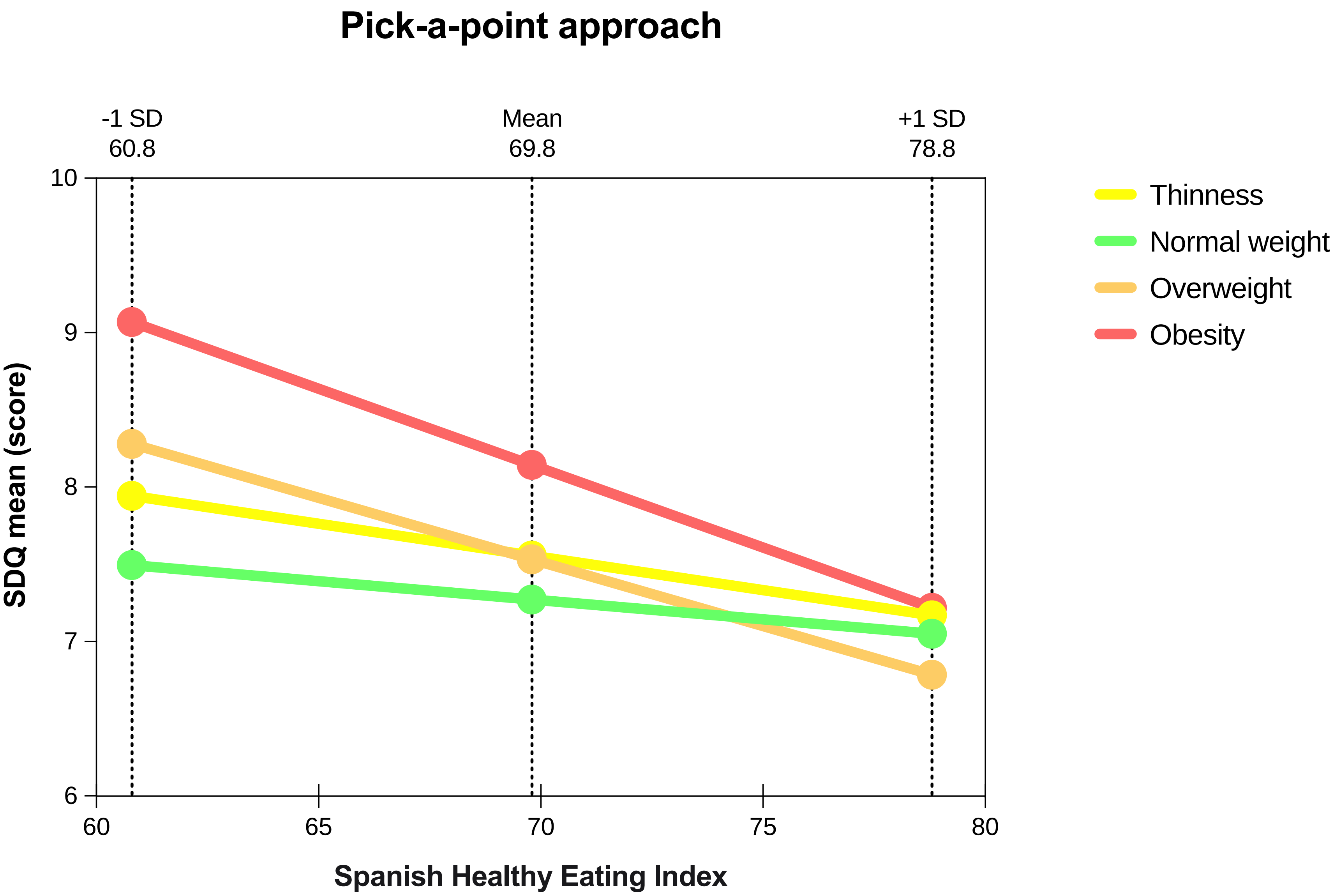

© 2022 López-Gil JF et al. *JAMA Network Open.*
